# Precursor B Lymphoblastic Lymphoma Involving the Stomach

**DOI:** 10.1155/2013/930918

**Published:** 2013-06-11

**Authors:** Masaya Iwamuro, Yoshinari Kawai, Yasuhide Yamawaki, Katsuyoshi Takata, Kazuhide Yamamoto

**Affiliations:** ^1^Department of Gastroenterology, Onomichi Municipal Hospital, Onomichi 722-8503, Japan; ^2^Department of Gastroenterology and Hepatology, Okayama University Graduate School of Medicine, Dentistry, and Pharmaceutical Sciences, Okayama 700-8558, Japan; ^3^Department of Internal Medicine, Onomichi Municipal Hospital, Onomichi 722-8503, Japan; ^4^Department of Pathology, Okayama University Graduate School of Medicine, Dentistry, and Pharmaceutical Sciences, Okayama 700-8558, Japan

## Abstract

Precursor B lymphoblastic lymphoma is a high-grade neoplasm arising from precursor lymphocytes of B-cell lineage. Extranodal sites such as the skin and bone are often involved, but gastrointestinal lesions of this disease are rarely encountered. Due to the infrequency, macroscopic forms of the gastrointestinal lesions have not been fully described. In this report, we present a case of precursor B lymphoblastic lymphoma involving the stomach, pancreas, bone, and bone marrow. Esophagogastroduodenoscopy showed multiple flat elevated lesions with irregular mucosa in the stomach.

## 1. Introduction

Precursor B lymphoblastic lymphoma is a neoplasm of lymphoblasts committed to the B-cell lineage [1]. Histologically, neoplastic cells are composed of small- to medium-sized lymphoid cells with blastic nuclear chromatin and a high mitotic rate [2]. In immunohistochemical analysis, the disease is characterized by positive staining for terminal deoxynucleotidyl transferase (TdT), in addition to B-cell antigens [1]. Clinically, extranodal sites such as the skin and the bone are frequently affected. On the other hand, infiltration of the neoplastic cells into the gastrointestinal tract is relatively infrequent. Therefore, the endoscopic manifestation of this disease has not been fully revealed to date.

We recently treated a patient with precursor B lymphoblastic lymphoma who had extensive extranodal involvement in the stomach, pancreas, bone, and bone marrow. Herein we report the case and illustrate its characteristic endoscopic features.

## 2. Case Presentation

An 85-year-old Japanese female presented to Onomichi Municipal Hospital with facial pain and lockjaw due to a swelling of the left temporomandibular joint. Her pain had started one year previously. The patient went to another clinic at that time, but the cause of her symptoms was not revealed. Two months prior to her visit to our hospital, she went to a dental clinic because lockjaw had appeared. Subsequently, swelling of the temporomandibular joint worsened and she was referred to our hospital for further investigation and treatment. She had been taking antihypertensive agents for hypertension and antifungal agent for nail tinea of the feet. The patient had no previous history of hematopoietic or gastrointestinal diseases. Physical examination revealed no abnormalities except for a mass lesion in the left temporomandibular joint. There was no evidence of hepatosplenomegaly or peripheral lymphadenopathy. Laboratory findings revealed elevated levels of lactate dehydrogenase (LDH) to 232 IU/L (normal range: 104–203 IU/L) and soluble interleukin-2 receptor (sIL-2R) to 1241 U/mL (normal range: 122–496 U/mL). Other laboratory findings were within normal ranges.

**Figure 1:**
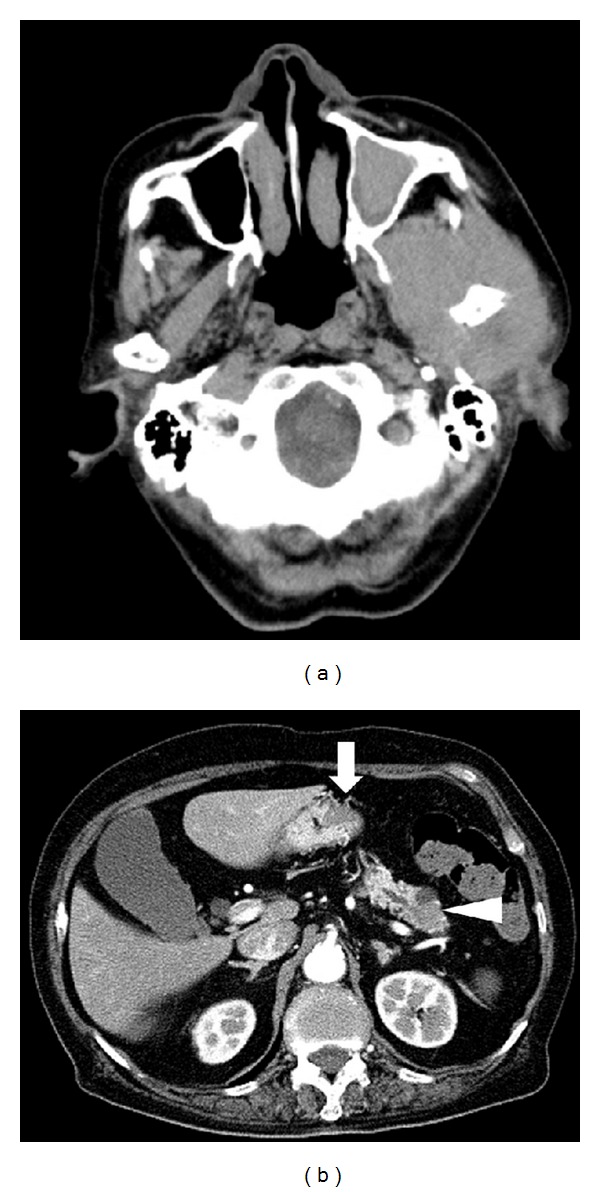
CT scanning images. A soft tissue density lesion 6 cm in diameter in the left temporomandibular joint is shown (a). Multiple hypovascular tumors were also detected in the stomach (arrow) and the pancreas (arrowhead) (b).

**Figure 2:**
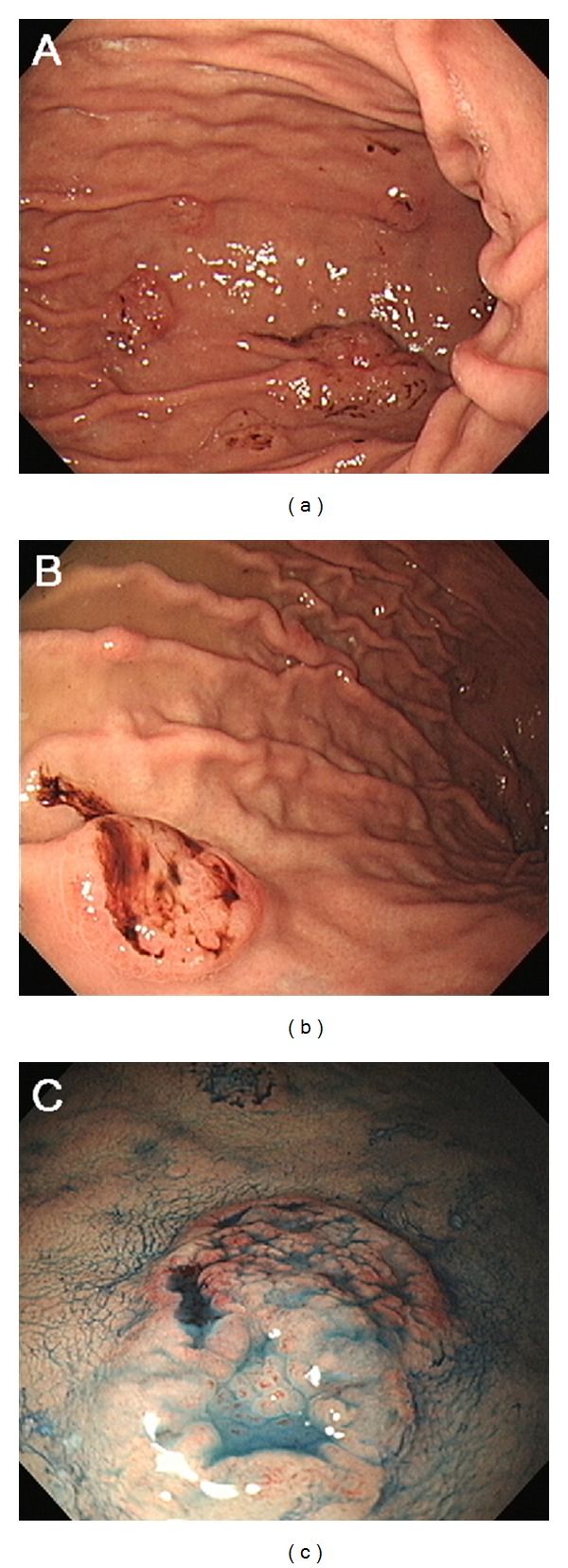
Endoscopic images of the stomach. Multiple flat elevated lesions with irregular and fragile mucosa are shown. Some of them had spontaneous bleeding (a, b). Indigo carmine contrast spray emphasized the irregular mucosa (c).

**Figure 3:**
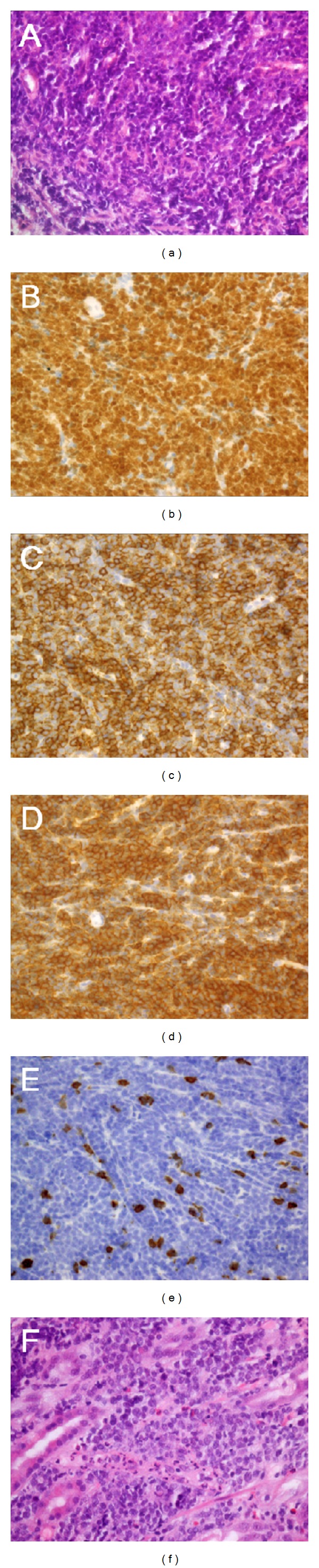
Histopathological images. Biopsy samples taken from the mass lesion in the temporomandibular joint revealed monotonous infiltration of medium-sized lymphoid cells with a high nuclear-cytoplasmic ratio ((a) hematoxylin and eosin staining, original magnification: ×40). These cells were positive for TdT (b, ×40), CD20 (c, ×40), and CD10 (d, ×40) but negative for CD3 (e, ×40). Neoplastic lymphoma cells were also detected in the stomach (f: hematoxylin and eosin staining, ×40).

Computed tomography (CT) scans of the head revealed a mass lesion with soft tissue density 6 cm in diameter in the left temporomandibular joint (Figure 1(a)). CT scans of the neck, chest, abdomen, and pelvis detected multiple hypovascular tumors in the stomach and the pancreas (Figure 1(b)). No lymphadenopathy was observed in the whole body. Esophagogastroduodenoscopy was performed and demonstrated multiple flat elevated lesions in the stomach (Figure 2). These lesions had irregular and fragile mucosa with redness; some of them showed spontaneous bleeding without contact of the endoscope. Biopsy samples taken from the mass lesion in the temporomandibular joint revealed a monotonous proliferation of medium-sized lymphoid cells with a high nuclear-cytoplasmic ratio and fine nuclear chromatins (Figure 3(a)). In immunohistochemical analysis, the neoplastic cells were positive for TdT, CD20, and CD10 but negative for CD3 (Figures 3(b)–3(e)). These cells were also detected in the biopsy samples from the gastric lesions (Figure 3(f)). Moreover, bone marrow aspirate and biopsy revealed infiltration of the neoplastic cells in the bone marrow. The tumor cells accounted for 4.4% of the marrow cells. Consequently, the patient was diagnosed with precursor B lymphoblastic lymphoma with bone marrow involvement, which formed multiple mass lesions in the head, stomach, and pancreas.

THP-COP chemotherapy (tetrahydropyranyl adriamycin, cyclophosphamide, vincristine, and prednisolone) in combination with rituximab was administered. In addition, radiotherapy was performed for palliation of her pain and lockjaw. However, the lymphoma lesions did not respond to the treatment. The patient deteriorated and died after 9 months from precursor B lymphoblastic lymphoma.

## 3. Discussion

Precursor B lymphoblastic lymphoma is a high-grade neoplasm arising from precursor lymphocytes of B-cell lineage [2, 3]. Approximately 75% of the patients with this disease are less than 18 years of age [1]. For example, Lin et al. reported that the median age of their 25 patients was 20 years [2]. In this case report, however, the patient was diagnosed at 85 years of age. Occurrence in such an elderly patient is uncommon in this disease.

Pathologically, typical features of precursor B lymphoblastic lymphoma are lymphoid cells of small to medium size with fine chromatin, inconspicuous nucleoli, and a high mitotic rate [4]. These neoplastic cells are positive for TdT and are almost always positive for B-cell antigens such as CD19 and CD79a [1, 5–7]. The lymphoblasts are also positive for CD10 and CD24 in most cases, whereas there is variable expression of CD20 and CD22. Histological and immunophenotypical features of precursor B lymphoblastic lymphoma are indistinguishable from those of precursor B lymphoblastic leukemia. Because of the biologic unity of the two disease entities, these tumors are considered to represent different clinical presentations of the same neoplasm, and the diagnosis is arbitrary to some extent. Patients with bone marrow involvement comprising more than 25% of the cellularity and/or patients with blood involvement are regarded as having precursor B lymphoblastic “leukemia.” On the other hand, patients with a mass lesion without any or with only minimal evidence of blood and marrow involvement are considered as precursor B lymphoblastic “lymphoma” [1]. In the present patient, the diagnosis of precursor B lymphoblastic lymphoma was made because the patient had mass lesions in the head, stomach, and pancreas, and the marrow involvement represented only 4.4% of the cellularity.

In patients with precursor B lymphoblastic lymphoma, the most frequently involved extranodal sites are the skin and the bone. Gastrointestinal tract can be affected, but such involvement has been described in only several case reports or as a part of case series [2, 8–12]. Therefore, because of the infrequency, macroscopic features of the gastrointestinal lesions of this disease have not been fully revealed. He et al. reported a patient with primary gastric precursor B lymphoblastic lymphoma who presented with a diffusely thickened gastric wall and large ulcer [8]. In the present patient, esophagogastroduodenoscopy showed multiple flat elevated lesions with irregular mucosa. To our knowledge, this is the second report presenting endoscopic images of the gastric involvement of this disease. In general, gastric lymphomas vary in morphology, for example, ulcerated, polypoid, granulonodular, and infiltrative [13]. Further evidence is required to determine whether gastric precursor B lymphoblastic lymphoma has a specific morphology that is distinguishable from other types of gastric lymphomas.

It is well known that multifocal lesions in the stomach, as described in our patient, are representative features of a gastric lymphoma, rather than a gastric cancer. Once a gastric lymphoma is suspected, endoscopists should perform biopsy and appropriate histopathological evaluation to specify lymphoma subtype. In the present patient, monomorphic proliferation of lymphoid cells with positive expression of TdT and B-cell markers led to a final diagnosis of precursor B lymphoblastic lymphoma.

In summary, we present a rare case of precursor B lymphoblastic lymphoma involving the stomach, pancreas, bone, and bone marrow. Esophagogastroduodenoscopy revealed multiple flat elevated lesions with irregular mucosa in the stomach.
